# Supplementary material for: Planning and commitment prompts to encourage reporting of HIV self-test results: A cluster randomized pragmatic trial in Tshwane District, South Africa
Source: PLOS Glob Public Health. 2022 Oct 24;2(10):e0001196. doi: 10.1371/journal.pgph.0001196 (PMC10021661; doi:10.1371/journal.pgph.0001196)
Supplement: S1 Text — (PDF) [file pgph.0001196.s002.pdf]

Behavioral nudges for HIV self-testing in South Africa
Plan and Pledge Study
IRB Protocol #833047
Version 2, May 3 2019

1. BACKGROUND AND STUDY RATIONALE

Reaching the "90-90-90" targets and ending the AIDS epidemic are bold targets that motivate current efforts in HIV prevention and treatment globally. Driven by large investments of resources, these efforts have included a rapid scale-up of HIV services, including HIV testing, medical male circumcision, and antiretroviral therapy (ART). However, behavioral factors have been important barriers to maximizing the effectiveness of resources devoted to HIV/AIDS. This is particularly evident in the low uptake of HIV services among young men in in eastern and southern Africa, with limited uptake of testing and linkage to HIV care in this population contributing to HIV incidence that remains above elimination targets. Insights from behavioral economics and related disciplines have highlighted various barriers to health-seeking behavior generally and also pointed to an array of low-cost interventions that have the potential to increase uptake of HIV services and promote behavior change more generally.

In collaboration with Wits Reproductive Health and HIV Institute (WRHI), University of the Witwatersrand, this pilot will incorporate behavioral insights to increase uptake of HIV self-testing in South Africa. The pilot will be part of WRHI's STAR program (HIV Self-Testing Africa) at the fixed-distribution sites. A copy of the approved protocol for the STAR program is included in this submission.

Further background on the HIV epidemic in South Africa and HIV self-testing can be found in the attached protocol for the STAR initiative. Background for this specific pilot includes the following:

Commitment devices and planning prompts are effective strategies for individuals to translate their behavioral intentions into actions, particularly when barriers to the desired behavior include procrastination, prospective memory failures, and hassle factors [1-5]. Planning prompts may also be effective in overcoming ambiguity aversion and information avoidance, and can leverage people's intrinsic desire to maintain a consistent self-image and also do what they believe to be the descriptive and injunctive norm [6-7].

In the context of the STAR program that is distributing HIV self-tests in South Africa, one of the desired outcomes is that individuals who obtain a self-test kit at distribution sites go on to take the test either immediately on site or in the near future at their home. A second desired outcome is that after using the self-test kit, individuals who use self-tests will report their results to a WhatsApp number that has been established for this purpose. A third desired outcome is that individuals who obtain a reactive test result will seek a confirmatory test at local clinics that provide HIV testing and counseling services. Those with a confirmed HIV-positive test result can be encouraged to seek HIV care, which includes treatment with antiretroviral therapy (ART). This pilot project will test whether commitment and planning prompts administered at the time of self-tests distribution are effective in promoting the desired behaviors.

1.1 Abbreviations

- STAR program: HIV Self-Testing Africa
- WRHI: Wits Reproductive Health and HIV Institute, University of the Witwatersrand

- HIVST: HIV self-testing
- UPenn: University of Pennsylvania

2. STUDY OBJECTIVES

2.1 Primary Objective

Determine the effectiveness of commitment and planning prompts on the likelihood that self-test recipients will report the use the HIV self-test kit on a dedicated WhatsApp number

2.2 Secondary Objectives

Determine the effectiveness of commitment and planning prompts on the likelihood that self-test recipients will 1) consent to phone/SMS follow-up, 2) Are successfully reached for follow-up by phone about 7 days after receiving the HIV self-test, 3) Report self-test result on the dedicated WhatsApp number and are HIV positive, 4) test on-site 5) Seek confirmatory testing

3. STUDY DESIGN

3.1 General Design

This study will use a cluster randomized trial design to test the effectiveness of interventions designed to increase usage of self-test and reporting of results by HIV self-tests recipients. The intervention will build on the current WhatsApp reporting instructions distributed during self-test distribution by the STAR program. A copy of these instructions are included in this submission. The two arms in the study will be: 1) **Usual Care, "Promote":** A card with information to report results via WhatsApp, similar to the existing card used in the STAR program. with a dedicated USUAL CARE WhatsApp number (different from the existing campaign numbers). 2) **Plan and Pledge:** The Usual Care card + a brief template to "make a plan" and "make a pledge" for test completion and results reporting, to take the test. The card will include a dedicated Plan and Pledge WhatsApp number.

Potential self-tester recipients will be shown the card, including Plan and Pledge statements if assigned to this condition, and will be encouraged by STAR field staff to use the card in their own time to make a plan and sign the pledge as part of receiving the test kit and instructions for how to complete the cards (see draft scripts below). Importantly, testers will be able to keep the card for themselves. There is no expectation to share the plan and pledge signature with the STAR field staff who distribute self-tests. Field staff will clarify for self-tests recipients that the Plan and Pledge process and card do not change the confidentiality of testing in any way.

Examples of script used for each arm:

Usual care, "Promote": On this card you'll see this WhatsApp number. After you do your screening test, you can report the test results to this number – it's very easy, and it's completely confidential. It's important to report your results – positive or negative – for three reasons: 1. So we know you used the test and it went ok. 2. So we can help you understand the results. 3. If you need our support to help you get care and services you need. If you don't have a camera on your phone, you can just text the results – see the instructions here. What questions do you have about reporting results?

Plan and Pledge: *Same as usual care, with the addition of:* On this part of the card, you have a place where you can make a plan to do the test – like when you'll do it, where, and if anyone will be with you when you do the test. This plan is just for you, we don't need to see it, but it's a good way to help you do the test now that you've picked it up. And then on this part of the card, you have a place where you can make a pledge or commitment to yourself to do the test and report results when you're done to the WhatsApp number. This pledge is just for you to help you carry out your plan to do the test and report results.

3.2 Randomization

We will use cluster randomization with team-day pair serving as the unit of randomization (i.e. two pairs of STAR field staff will be randomized by arm and by day. Clusters of individuals will receive self-tests at a distribution site from a pair of STAR field staff on an individual day). Computer-generated randomization will be used to assign team-day pair clusters to the 2 study arms. Sites will the STAR community fixed-point distribution sites in Tshwane District, South Africa.

3.3 Study Measures

The study will be implemented by WRHI. UPenn will only partake in the design and the analysis of de-identified aggregate data. As such, all study procedures will be conducted by the WRHI team and reviewed by the University of Witwatersrand IRB. WHRI will share any modifications submitted to Witwatersrand IRB with the UPenn team, and the UPenn team will share these documents with UPenn IRB according to the specific requirements and procedures of the UPenn IRB.

During distribution of the self-tests, an intake form will be completed (a copy of this form can be found in the attached STAR SOP). This form will track the self-test recipient's arm assignment, on-site of off-site testing, and consent for follow-up phone call.

If a self-test recipient messages the WhatsApp number or receive a follow-up call, they will be asked the following series of questions (questions are sequential and continuation of questions is dependent on response to previous question): Did they use self-test? What is their test result? (if they are willing to share), Would like a confirmatory test? Did they attended confirmatory testing? What was the confirmatory test result? Would they want to be linked to care? What date were they linked to care? ART initiation? Date of ART initiation? Linked to VMMC? Date of VMMC?

As outlined in the attached STAR WhatsApp card, all text messages are confidential and de-identified. Data tracking record keeping is also de-identified with only the HIV self-test kit bar code recorded.

Please see section 5 Statistical Plan for outcomes and analysis.

STUDY POPULATION AND DURATION OF PARTICIPANTION

3.4 Duration of Study Participation

The intervention will be implemented at the time of HIV self-test distribution and there is no follow-up period. Participants are instructed to use the HIV self-test at home and complete the Plan and Pledge form whenever they choose. If the participants opts to test on-site, they can complete the Plan and Pledge on-site as well. Participants are asked during distribution if they would like to receive a follow-up call, as part of standard procedures with the STAR program. Follow-up calls will be made by the WRHI team about 5 days after distribution. Follow-up calls may include, if the individual used the HIV self-test, if they are willing to share their test result, and questions about the Plan and Pledge intervention, such as if the individual completed the Pledge and Plan cards.

3.5 Total Number of Subjects and Sites

Our target enrollment will be approximately 1,320 self-test recipients, with about 660 recipients per arm. Project site will be the Tshwane district, South Africa.

3.6 Inclusion and Exclusion Criteria

Please reference the attached STAR protocol for inclusion and exclusion criteria. This study will be implemented by WHRI as part of the STAR program and will follow the same inclusion and exclusion criteria.

Inclusion: Must be 19 years or older

Exclusion: Any potential testers that are visibly intoxicated will be turned away, as alcohol effects the test results.

3.7 Subject Recruitment

The WHRI field team will set-up a distribution tent in study site areas. Individuals walking or driving past can decide to stop by the tent to learn more about the HIV self-test and to collect for personal use. Individuals in the general area of the distribution site can self-select or volunteer to collect a HIV self-test.

3.8 Vulnerable Populations

This study will not recruit vulnerable populations such as children or prisoners. The STAR program follows the South Africa National Department of Health guidelines for HIVST. According to these guidelines, pregnant women are not excluded from collecting an HIV self-test.

Collection of HIV self-tests, testing, and reporting of test results to study WhatsApp number are all optional. Participants can opt to not complete any of the testing or follow-up steps at any time. This also applies to the Pledge and/or Plan cards.

4. STUDY PROCEDURES

Please reference the STAR protocol and SOP for study procedures. The UPenn study team will be part of study design and analysis of de-identified aggregate data but will not play any role in study implementation. All aspects of implementation will be managed by WRHI and the University of Witwatersrand IRB.

5. STATISTICAL PLAN

5.1 Sample Size and Power Determination

To detect a difference in the primary outcome of 5% percentage points (from 2.5% reporting to 7.5% reporting) with 80% power and at alpha = .05, with rho=.01 and cluster size per day = 30 per arm, we plan to recruit 660 participants per arm with a total of 1,320 participants.

5.2 Statistical Methods

We will compare primary and secondary outcomes in the Plan and Pledge arm to the Usual Care, "Promote" arm using 2-tailed chi-squared tests as well as logistic regression analyses. Analyses will adjust for correlation of outcomes within clusters. We hypothesize that a higher proportion of participants in the Plan and Pledge arm will report their self-test results than in the Usual Care arm, "Promote".

5.2.1 Analysis of Primary Outcome

Our primary outcome is the reporting self-test usage, measured by the percent of self-test recipients reporting use of HIV self-test kits to dedicated WhatsApp number by arm and compared to the standard of care arm of the STAR program

5.2.2 Analysis of Secondary Outcome(s)

1) The percent of self-test recipients who consent to phone/SMS follow-up by study arm and compared to the standard of care arm of the STAR program. 2) The percent of self-test recipients who are successfully reached for follow-up by phone about 7 days after receiving the HIV self-test, by study arm and compared to the standard of care arm of the STAR program, 3) The percent of self-test recipients who report self-test result on the dedicated WhatsApp number and are HIV positive, by study arm and compared to the standard of care arm of the STAR program. 4) The Ratio of negative:positive test results reported by study arm and compared to the standard of care arm of the STAR program.. 5) Percent of on-site testers by study arm and compared to the standard of care arm of the STAR program. 6) The percent of self-test recipients who seek confirmatory testing by study arm and compared to the standard of care arm of the STAR program

6. SAFETY AND ADVERSE EVENTS

The study will be implemented by the WRHI team. UPenn will only partake in the design and the analysis of de-identified aggregate data. As such, any adverse events will be reported by the WRHI team to the University of Witwatersrand IRB, and will be handled according to their specific procedures. WHRI will share any AEs submitted to Witwatersrand IRB with the UPenn team, and the UPenn team will share these documents with UPenn IRB according to the specific requirements and procedures of the UPenn IRB.

7. STUDY ADMINISTRATION, DATA HANDLING, AND RECORD KEEPING

The study will be implemented by the WRHI team. UPenn will only partake in the design and the analysis of aggregate de-identified data. Therefore all study administration, data handling, and record keeping, will be managed by WRHI. WRHI will manage all communications and documentation with the local IRB at the University of Witwatersrand. Any changes to the "Plan and Pledge" pilot will be submitted to the UPenn IRB for review. De-identified aggregate data will be shared with the UPenn team through secure channels (password protected and/or encrypted).

8. STUDY MONITORING

The study will be monitored by the PI and managers at WRHI, as listed on the STAR protocol.

9. ETHICAL CONSIDERATIONS

9.1 Risk

Based on the current and on-going HIV self-test distribution by WRHI and STAR program, we believe that the study is minimal risk. As with any HIV testing research there is the possibility of psychological distress for study participants due to testing. To minimize risk the STAR program diligently follows the HIVST guidelines of South Africa provided by the National Department of Health.

9.2 Benefits

The main direct benefit of this study, is that we will provide free HIV self-testing kits, allowing individuals to know their HIV status in the privacy of their home. Indirectly, the study may give rise to increased identification of HIV-positive persons and increased engagement in HIV care and treatment, thereby reducing the overall number of HIV transmissions occurring as well.

9.3 Risk and Benefit Assessment

We believe that the benefits of this study far outweigh the risks. Given the public health importance of achieving higher uptake of HIV testing among high-risk populations, it is vital to find effective new strategies that can be used to promote testing and identify HIV-positive persons unaware of their HIV infection. The knowledge gained from this study is particularly relevant at a time when countries in Africa are actively developing HIV self-testing policies and seeking optimal ways to scale-up HIV self-tests. Our study will provide data on low-touch, inexpensive nudges, than can be added to currently existing HIVST distribution programs.

9.4 Informed Consent Process

We are asking for a waiver in consent. As outlined in section 5.1 and 5.4, involvement in the study will be voluntary. Participation will be opt-in and it is likely that the act of providing informed consent will influence the effect of the interventions and outcomes in the usual care group.

Study consent procedures will follow the approved STAR program protocol in which self-test recipients provide verbal consent to receiving follow-up calls to ascertain whether they used self-tests and self-test results.

10. STUDY FINANCES

10.1 Funding Source

The STAR program with WRHI is funded by UNITAID and the Gates Foundation. The UPenn pilot is funded by the Gates Foundation, grant number OPP1195218.

10.2 Conflict of Interest

No COI to report. All University of Pennsylvania Investigators will follow the University of Pennsylvania Policy on Conflicts of Interest Related to Research.

11. PUBLICATION PLAN

Findings from this study will be published in peer-reviewed journals and presented at scientific conferences. There is no projected impact on publication or presentation plans.

12. REFERENCES

1. Bryan, G., Karlan, D., & Nelson, S. (2010). Commitment devices. Annu. Rev. Econ., 2(1), 671-698.

2. Rachlin H. Self-control based on soft commitment. The Behavior Analyst. 2016 Oct 1;39(2):259-68.

3. Grieco D, Lacetera N, Macis M, Martino D. Motivating Cord Blood Donation with Information and Behavioral Nudges. Scientific reports. 2018 Jan 10;8(1):252.

4. Rogers T, Milkman KL, Volpp KG. Commitment devices: using initiatives to change behavior. JaMa. 2014 May 28;311(20):2065-6.

5. Jessica Cogen et al. Measuring the impact of cash transfers and behavioral 'nudges' on maternity care in Nairobi, Kenya. 2017. Health Affairs 26(11).

6. Rogers T, Milkman KL, John LK, Norton MI. Beyond good intentions: Prompting people to make plans improves follow-through on important tasks. Behavioral Science & Policy. 2015;1(2):33-41.

7. Milkman KL, Beshears J, Choi JJ, Laibson D, Madrian BC. Using implementation intentions prompts to enhance influenza vaccination rates. Proceedings of the National Academy of Sciences. 2011 Jun 28;108(26):10415-20.

13. ATTACHEMNTS

1. STAR protocol
2. STAR protocol ethics approval
3. STAR program fixed distribution SOP
4. STAR program WhatsApp card
5. Plan and pledge commitment card
