# Supplementary material for: Planning and commitment prompts to encourage reporting of HIV self-test results: A cluster randomized pragmatic trial in Tshwane District, South Africa
Source: PLOS Glob Public Health. 2022 Oct 24;2(10):e0001196. doi: 10.1371/journal.pgph.0001196 (PMC10021661; doi:10.1371/journal.pgph.0001196)
Supplement: Attachment — Submitted filename: Response to Reviewers.docx [file pgph.0001196.s003.docx]

**Response to Reviewers**

Journal Requirements:

1. Please amend your detailed Financial Disclosure statement. This is published with the article. It must therefore be completed in full sentences and contain the exact wording you wish to be published.

State the initials, alongside each funding source, of each author to receive each grant.

**Mention of funding was removed from the manuscript. On the portal, we included the following statement based on the** [**journal submission guidelines.**](https://journals.plos.org/globalpublichealth/s/submission-guidelines#loc-financial-disclosure-statement)

***This study was funded by the Bill & Melinda Gates Foundation (gatesfoundation.org) through award INV-008318 (HT). The funders had no role in study design, data collection and analysis, decision to publish, or preparation of the manuscript. No conflicts of interest were declared.***

2. In the online submission form, you indicated that your data will be submitted to a repository upon acceptance. We strongly recommend all authors deposit their data before acceptance, as the process can be lengthy and hold up publication timelines. Please note that, though access restrictions are acceptable now, your entire data will need to be made freely accessible if your manuscript is accepted for publication. This policy applies to all data except where public deposition would breach compliance with the protocol approved by your research ethics board. If you are unable to adhere to our open data policy, please kindly revise your statement to explain your reasoning and we will seek the editor's input on an exemption. Please be assured that, once you have provided your new statement, the assessment of your exemption will not hold up the peer review process.

**We are also committed to depositing de-identified data to the OSF repository and will complete this process promptly in accordance with our protocol. We are familiar with this process and do not anticipate lengthy delays.**

3. Please provide separate figure files in .tif or .eps format and ensure that all files are under our size limit of 10MB.

For more information about how to convert your figure files please see our guidelines: <https://journals.plos.org/globalpublichealth/s/figures>.

**Both Figures have been converted to .tif and are under 10MB.**

4. We have noticed that you have uploaded Supporting Information files, but you have not included a list of legends. Please add a full list of legends for your Supporting Information files after the references list.

**We apologize for any confusion with our original submission. We have 1 table and 2 figures in our manuscript, and no additional Supporting Information. We have clarified the placement of tables and figures in the manuscript and the labeling of Figure files and captions.**

Additional Editor Comments (if provided):

Reviewer #1: This study which investigated an important question relating to action following self-testing for HIV is very interesting. While the research question is very relevant, I found a couple of pieces not speaking to the correct design.

1. The study is given as a cluster randomized controlled trial yet the sample size calculation and the analysis is done at individual level.

**We have added text to clarify that our power calculations do account for the clustered design. Due to constraints in our ability to match test results reports to clusters, we are not able to adjust confidence intervals for clustering. We have revised the text to clarify this.**

2. The manuscript does not follow any reporting guidelines such as the CONSORT Extension for cluster randomized trials.

**We prepared the manuscript following the CONSORT guidelines and have included a completed checklist with the revision, as we did in the original submission.**

3. The results section is simply too brief.

**This pragmatic trial has a very straightforward outcome which can be succinctly described (as well as shown graphically), following editorial guidance to “present and discuss findings concisely.” Addressing other reviewer concerns, we have also provided some demographic characteristics of the two intervention groups and include a brief description of this table in the Results section. We have also added an exploratory comparison of response to the interventions by gender.**

4. The outcome measurement is not properly given.

**We are not exactly sure what the reviewer’s concern is here but have revised the description of the primary and secondary outcomes for better clarity and precision. They now read: “The primary outcome was test results reported to the program’s WhatsApp numbers as a proportion of tests distributed. The secondary outcome was the proportion of HIV-positive test results reported as proportion of tests distributed (i.e., reported yield).”**

5. The study population is not completely described i.e. the inclusion and exclusion criteria is not well described.

**We have included more information about the study population by adding more information about the distribution sites, and the inclusion and exclusion criteria. We have also added a Table of sociodemographic characteristics of the two intervention groups.**

6. The comparator is not very clear: is it the enhanced SOC or just SOC and how the implementation was done may have biased results because the authors say that they did intervention first and SOC later on each day.

**Thank you for this comment. The main comparison is the Plan & Commit condition to the Enhanced Usual Care. This is stated clearly in the Abstract and is discussed at length in the Materials and Methods section under *Interventions* and *Analysis* subsections. We have also added language in the Analysis section to restate that the primary preregistered analysis is the comparison between Plan & Commit and Enhanced Usual Care, and that the comparison of both interventions to the exiting STAR protocol is an exploratory analysis. The implementation of the Plan & Commit and Enhanced Usual Care conditions in the morning should not bias this primary comparison, as these two conditions were both implemented at this start of the testing day. While potential testers who stopped by the distribution site later in the day may have been systematically different from those arriving earlier in the day, we also note that many of the test recipients the exploratory analysis received their tests at different sites from the intervention sites and would therefore have also been able to receive standard protocol tests and counseling first thing in the morning. While we are not concerned about this potential source of bias, we have noted it in the limitations section.**

Reviewer #2: A well written manuscript which addresses an important topic of results reporting around HIVST. It has been well established that HIVST does work in finding hard to reach populations, but linkage and results reporting remains a problem as rightly highlighted by the author. Even though there was a rise in the results reported in the intervention arms as compared to the standard arm, it would have been great if the author gave us more information on the characteristics of the participants. I suggest an addition of 2 tables, 1 for characteristics of those who took kits and another for those who reported results. In this way, a comparison can be made to see if there is a difference between those who did and those who did not report results which is important in the context of who we should expect to respond to these interventions.

**Thank you for this suggestion. We have added a Table 1 with a limited number of available characteristics for those who received tests and those who reported results back via WhatsApp for the Plan & Commit and the Enhanced Usual Care Conditions.**

While revising your submission, please upload your figure files to the Preflight Analysis and Conversion Engine (PACE) digital diagnostic tool, https://pacev2.apexcovantage.com/. PACE helps ensure that figures meet PLOS requirements. To use PACE, you must first register as a user. Registration is free. Then, login and navigate to the UPLOAD tab, where you will find detailed instructions on how to use the tool. If you encounter any issues or have any questions when using PACE, please email PLOS at figures@plos.org. Please note that Supporting Information files do not need this step.

**Figure 1 and Figure 2 were uploaded to PACE and the compatible file formats have subsequently been uploaded to the portal with the revision.**
